# Descriptive epidemiology of coronavirus disease 2019 in Nigeria, 27 February–6 June 2020

**DOI:** 10.1017/S095026882000206X

**Published:** 2020-09-11

**Authors:** K. O. Elimian, C. L. Ochu, E. Ilori, J. Oladejo, E. Igumbor, L. Steinhardt, J. Wagai, C. Arinze, W. Ukponu, C. Obiekea, O. Aderinola, E. Crawford, A. Olayinka, C. Dan-Nwafor, T. Okwor, Y. Disu, A. Yinka-Ogunleye, N. E. Kanu, O. A. Olawepo, O. Aruna, C. A. Michael, L. Dunkwu, O. Ipadeola, D. Naidoo, C. D. Umeokonkwo, A. Matthias, O. Okunromade, S. Badaru, A. Jinadu, O. Ogunbode, A. Egwuenu, A. Jafiya, M. Dalhat, F. Saleh, G. B. Ebhodaghe, A. Ahumibe, R. U. Yashe, R. Atteh, W. E. Nwachukwu, C. Ezeokafor, D. Olaleye, Z. Habib, I. Abdus-Salam, E. Pembi, D. John, U. J. Okhuarobo, H. Assad, Y. Gandi, B. Muhammad, C. Nwagwogu, I. Nwadiuto, K. Sulaiman, I. Iwuji, A. Okeji, S. Thliza, S. Fagbemi, R. Usman, A. A. Mohammed, O. Adeola-Musa, M. Ishaka, U. Aketemo, K. Kamaldeen, C. E. Obagha, A. O. Akinyode, P. Nguku, N. Mba, C. Ihekweazu

**Affiliations:** 1 Prevention, Programmes and Knowledge Management, Nigeria Centre for Disease Control, Abuja, Nigeria; 2 Department of Microbiology, Faculty of Life Sciences, University of Benin, Edo State, Nigeria; 3 Nigeria COVID-19 Research Consortium, Abuja, Nigeria; 4 Surveillance and Epidemiology, Nigeria Centre for Disease Control, Abuja, Nigeria; 5 Health Emergency Preparedness and Response, Nigeria Centre for Disease Control, Abuja, Nigeria; 6 School of Public Health, University of the Western Cape, Cape Town, South Africa; 7 Centers for Disease Control and Prevention, U.S. Embassy Abuja, Abuja, Nigeria; 8 Georgetown University, Abuja, Nigeria; 9 Public Health Laboratory Services, Nigeria Centre for Disease Control, Abuja, Nigeria; 10 World Health Organization, Abuja, Nigeria; 11 Ahmadu Bello University, Zaria, Nigeria; 12 African Field Epidemiology Network, Abuja, Nigeria; 13 Nigeria Field Epidemiology and Laboratory Training Program, Abuja, Nigeria; 14 Public Health England's International Health Regulations Strengthening Project, Abuja, Nigeria; 15 Tony Blair Institute for Global Change, Abuja, Nigeria; 16 University of Ilorin, Ilorin, Nigeria; 17 Department of Community Medicine, Alex Ekwueme Federal University Teaching Hospital, Abakaliki, Ebonyi State, Nigeria; 18 Resolve to Save Lives, Abuja, Nigeria; 19 National Agency for the Control of AIDS, Abuja, Nigeria; 20 Department of Virology, College of Medicine, University of Ibadan, Ibadan, Nigeria; 21 University of Abuja Teaching Hospital, Abuja, Nigeria; 22 Ministry of Health, Lagos State, Nigeria; 23 Ministry of Health and Human Services, Adamawa State, Nigeria; 24 Department of Public Health, Health and Human Services Secretariat, FCT, Abuja, Nigeria; 25 Edo State Ministry of Health, Edo State, Nigeria; 26 Ministry of Health, Kebbi State, Nigeria; 27 Department of Disease Control and Immunisation, Bauchi State Ministry of Health, Bauchi, Nigeria; 28 Department of Public Health, Kano State Ministry of Health, Kano State, Nigeria; 29 Department of Public Health, Abia State Ministry of Health, Abia State, Nigeria; 30 Department of Public Health, Rivers State Ministry of Health, Rivers State, Nigeria; 31 Department of Epidemiology and Disease Control, Katsina State Ministry of Health, Katsina, Nigeria; 32 Epidemiology Unit, Department of Public Health, Ministry of Health, Bayelsa State, Nigeria; 33 Epidemiology Unit, Department of Public Health, Ministry of Health, Imo State, Nigeria; 34 Epidemiology Unit, Department of Public Health, Ministry of Health, Borno State, Nigeria; 35 Department of Epidemiology and Disease Control, Ministry of Health, Ondo State, Nigeria; 36 Department of Public Health, Zamfara State Ministry of Health, Zamfara State, Nigeria; 37 Department of Public Health Services, Ministry of Health, Sokoto State, Nigeria; 38 Department of Public Health, Ministry of Health, Osun State, Nigeria; 39 Department of Public Health, Yobe State Primary Healthcare Management Board, Yobe State, Nigeria; 40 Department of Public Health, Taraba State Ministry of Health, Taraba State, Nigeria; 41 Department of Public Health, Kwara State Ministry of Health, Kwara State, Nigeria; 42 Anambra State Ministry of Health, Anambra State, Nigeria; 43 Directorate of Public Health, Oyo State Ministry of Health, Oyo State, Nigeria; 44 Office of the Director General, Nigeria Centre for Disease Control, Abuja, Nigeria

**Keywords:** Asymptomatic, case fatality, COVID-19, cumulative incidence, epidemiology, Nigeria

## Abstract

The objective of this study was to describe the epidemiology of COVID-19 in Nigeria with a view of generating evidence to enhance planning and response strategies. A national surveillance dataset between 27 February and 6 June 2020 was retrospectively analysed, with confirmatory testing for COVID-19 done by real-time polymerase chain reaction (RT-PCR). The primary outcomes were cumulative incidence (CI) and case fatality (CF). A total of 40 926 persons (67% of total 60 839) had complete records of RT-PCR test across 35 states and the Federal Capital Territory, 12 289 (30.0%) of whom were confirmed COVID-19 cases. Of those confirmed cases, 3467 (28.2%) had complete records of clinical outcome (alive or dead), 342 (9.9%) of which died. The overall CI and CF were 5.6 per 100 000 population and 2.8%, respectively. The highest proportion of COVID-19 cases and deaths were recorded in persons aged 31–40 years (25.5%) and 61–70 years (26.6%), respectively; and males accounted for a higher proportion of confirmed cases (65.8%) and deaths (79.0%). Sixty-six per cent of confirmed COVID-19 cases were asymptomatic at diagnosis. In conclusion, this paper has provided an insight into the early epidemiology of COVID-19 in Nigeria, which could be useful for contextualising public health planning.

## Introduction

On 31 December 2019, a cluster of cases of pneumonia of unknown aetiology was detected in Wuhan City, Hubei Province, China [1]. On 7 January 2020, the Chinese authorities identified and announced a novel type of coronavirus as the cause of the disease [2]. On 30 January 2020, the World Health Organization (WHO) declared the 2019-nCoV outbreak a Public Health Emergency of International Concern [3] and a few days later announced the official name of the virus as Severe Acute Respiratory Syndrome Coronavirus 2 (SARS-CoV-2) and the disease as Coronavirus Disease 2019 (COVID-19) [4]. COVID-19 was declared a pandemic on 11 March 2020 by the WHO.

The first case of COVID-19 in Nigeria was confirmed on 27 February 2020. The case was a 44-year old Italian citizen who arrived Nigeria through the Murtala Mohammed International Airport, Lagos, on a flight via Milan, Italy [5]. This index case led to the activation of COVID-19 Public Health Emergency Operation Centers (PHEOC) at national and sub-national levels, with associated active case finding via contact tracing. By 9 March 2020, 217 contacts were linked to this index case [5], out of which 136 (63.0%) were under follow-up, with one contact confirmed positive [6]. The 14-day follow-up for contacts of the index case ended on 12 March 2020. During this period, two additional unlinked cases were reported in Nigeria. In addition, 42 suspected cases were identified across seven states in Nigeria namely the Federal Capital Territory (FCT), Edo, Kano, Lagos, Ogun, Rivers and Yobe [5].

Since the confirmation of the first COVID-19 case in Nigeria, cases and deaths have risen steadily in the country, although the government has implemented public health interventions – e.g. advocacy for physical distancing, complete and partial lockdown, and ban on large public gatherings including at churches and mosques – to contain or mitigate spread. As of 6 June 2020, 35 (out of 36) states, plus the FCT, have reported at least one confirmed COVID-19 case. A descriptive analysis of the clinical characteristics, treatment modalities and outcomes of the first 32 COVID-19 patients admitted to Mainland Hospital in Lagos State, Nigeria, found that two-thirds of patients were male, and the mean age was 38.1 years [7]. This early analysis however is insufficient to provide a national overview of COVID-19 epidemiology in Nigeria. The Nigeria Centre for Disease Control (NCDC) coordinates the public health response to COVID-19 in the country. Through NCDC's surveillance and laboratory network as well as coordination of state PHEOCs, epidemiological information on COVID-19 cases are captured into a real-time networked platform called Surveillance Outbreak Response Management and Analysis System (SORMAS). This forms the basis for the release of daily situation reports for COVID-19 on NCDC COVID-19 microsite [8]. By 6 June, thousands of individual records with laboratory diagnosis contained on SORMAS offered opportunities to expand and explore country-specific epidemiologic and clinical characteristics of COVID-19 from the onset of the outbreak. This study aims to provide the initial descriptive epidemiology of COVID-19 in Nigeria, with emphasis on the disease magnitude and patterns in terms of person, place and time.

## Methods

### Study design, period and settings

We conducted a retrospective analysis of Nigeria surveillance data between 27 February and 6 June 2020. Nigeria is administratively divided into 36 states plus the FCT, which are zoned across six geopolitical areas: South-South; South-West; South-East; North-East; North-West and North-Central. During the study period, 36 states plus FCT had reported confirmed COVID-19 cases; all states were actively monitoring for cases through the Integrated Disease Surveillance and Response system (IDSR) system [9].

### Data source

SORMAS, an open-source real-time electronic health surveillance database, was the primary data source for this study. In 2017, NCDC adopted SORMAS as its primary digital surveillance platform for implementing the IDSR system [9], and customised it for the surveillance of priority diseases of public health importance in Nigeria. As part of the country's preparedness activities, a COVID-19 module was developed and added to SORMAS in January 2020. All the surveillance data generated through SORMAS is owned by NCDC, processed and stored in a central server at the NCDC headquarters in Abuja, Nigeria.

### Study population and data collection

The study population was persons investigated for SARS-CoV-2 infection and captured on SORMAS during the study period. Samples were collected from suspect cases in line with the NCDC case definitions (which were in turn derived from WHO case definitions) in Table 1 [10]. However, these guidelines were not strictly adhered to as samples were also collected from some asymptomatic cases and contacts of cases. Trained healthcare personnel (and rapid response team members) investigated suspected COVID-19 cases, completed a detailed case investigation form (CIF) and collected a minimum of one nasopharyngeal or nasal swab, and one oropharyngeal swab using synthetic fibre swabs with plastic shafts. Collected specimens were triple-packaged and aseptically transported in viral transport media, under appropriate temperature conditions (2–4 °C) to a designated NCDC-certified laboratory in the country, usually based on proximity. Laboratory diagnosis of COVID-19 was done by real-time polymerase chain reaction (RT-PCR) in accordance with the WHO interim guidelines [11]. In addition to clinical samples, information on patients' sociodemographic characteristics, signs and symptoms in the 14 days prior to diagnosis, laboratory findings and clinical outcome as detailed in the national CIF was captured on SORMAS. Surveillance and laboratory data were submitted by trained data collectors (i.e. healthcare personnel) in real time to the NCDC through the SORMAS platform (configured on mobile devices (e.g. tablets and smartphones) and laptops) by each reporting State Epidemiologist and testing laboratory, respectively. All laboratory-confirmed COVID-19 cases were managed according to the NCDC case management protocol [10], while adherence to infection prevention and control measures for both health workers and patient was ensured. Testing for COVID-19 during this study period is free of charge in Nigeria.

**Table 1.** Definition of key study variables

Variable	Variable definition and classification
Sociodemographic and clinical variables	
Suspect case^a^	Symptoms with international travel: Any person with acute respiratory illness (fever and either cough, difficulty breathing or shortness of breath) OR new respiratory symptoms without fever (cough, difficulty breathing or shortness of breath) and no other explanation, AND a history of travel to or residence in a country reporting COVID-19 within 14 days prior to symptom onset; OR Symptoms with contact to confirmed case: Any person with new respiratory symptoms (cough, difficulty breathing or shortness of breath, with or without fever), AND had contact with a confirmed or probable COVID-19 case (see definition of contact) in the last 14 days prior to symptom onset; OR Acute respiratory illness in an area of moderate or high COVID-19 prevalence with no other explanation: Any patient with acute respiratory illness within the last 10 days (fever and either cough, difficulty breathing or shortness of breath); AND in absence of an alternative diagnosis that explains the clinical presentation AND residing or working in the last 14 days in an area identified by NCDC as a moderate or high prevalence region.
Probable case	Any individual who met the criteria for a suspect case and for whom testing for COVID-19 was inconclusive or for whom testing was positive on a pan-coronavirus assay or where samples were not collected before the demise of a suspect case.
Confirmed case	Any individual with laboratory confirmation of SARS-CoV-2 infection with or without signs and symptoms.
Non-case	A non-case was defined as an individual whose RT-PCR test was negative for SARS-CoV-2.
Contact^b^	Any individual who had contact (within 1 m for at least 15 min) with a confirmed case during their symptomatic period, including one day before symptom onset.
Clinical outcome	Clinical outcome was classified as a binary variable: survivor and death. A survivor was a COVID-19 case who was officially discharged after two consecutive negative tests for SARS-CoV-2; however, one negative test as discharge criterion was implemented on 2 May 2020.
Sex	Sex was defined as either male or female.
Age	Age, in years, was based on self-reports by a person or a relative, and was treated both as continuous and categorical variables, depending on the study priority. As a categorical variable, age was classified on the basis of clinical and public health relevance as well as for ease of interpretation: 0–4; 5–13; 14–20; 21–30; 31–40; 41–50; 51–60; 61–70; 71–80; >80.
Residential setting^c^	Residential setting of each person tested for COVID-19 was based on the population size and administrative/legal criteria for the reporting Local Government Areas (LGA) as recorded by field staff, in line with common classification of urban and rural classification in Nigeria [12]. For example, an LGA was classified as urban if ‘any one’ of the following criteria was met: (1) State capital; (2) an estimated population size of ⩾20 000; (3) >75% of its population is engaged in non-agricultural occupations; (4) availability of infrastructure, good transportation system and a broad array of economic, social and recreational activities.
Health facility	Health facility refers to the type of facility each person tested for COVID-19 visited prior to diagnosis or was identified for diagnosis. Because it was listed on SORMAS without specific categorisation into health facility type, we utilised the Nigeria Health Facility Registry (HFR) of the Federal Ministry of Health [13] to identify each health facility type so as to minimise misclassification errors. The HFR has details of all the registered health facilities in Nigeria including the State, LGA, facility level (primary, secondary and tertiary) and ownership type (private and public). Overall, each health facility was defined either as primary, secondary, or tertiary facility; health facilities that could not be identified in the registry were treated as unknown.
Education completed	Classified as a categorical variable in line with the Nigerian educational system: No formal education; nursery/primary; secondary and tertiary/post-secondary. However, given the peculiar nature of the Almajiranci/Quranic educational system in Nigeria, they were classified under a separate category termed ‘alternative’ education.
Current occupation	Classified as a categorical variable as follows: Pupil/student; child; housewife; trader/business; health professional (e.g. nurse, clinician, laboratorian etc.); animal-related work (e.g. butcher and hunter); farmer; religious/traditional leaders; transporter and other.
Travel history	Classified as local, international and no travel in the last 14 days prior to diagnosis.
Clinical signs and symptoms	Defined relative to 14 days before sample collection and classified as binary: yes/no. Examples of clinical variables include fever (defined as an axillary temperature of 37.5 °C or higher), cough, difficulty breathing, diarrhoea, headache among others.
Quarantine location	Defined as a binary variable: formal institution (e.g. health facility) and informal institution (e.g. home).
Time variables (in days)^d^	
Time from symptom onset to diagnosis	Defined as the time difference between the dates of sample collection and self-reported symptom onset among symptomatic COVID-19 cases only.
Duration in quarantine	Defined as the time difference between the end and start dates of quarantine; it was treated as a continuous variable.
Time to hospitalisation	Defined as the time difference between the date of hospital visit or admission and date when laboratory result was ready; it was treated as a continuous variable.
Duration of hospitalisation	Defined as the time difference between the dates of discharge/transfer and visit/admission to hospital; it was treated as a continuous variable.
Time to death from diagnosis	Defined as the time difference between the dates of death and sample collection for laboratory diagnosis of COVID-19; it was treated as a continuous variable.
Time from sample collection to arrival in the laboratory	Defined as the time difference between the dates of sample arrival in the laboratory and sample collection; it was treated as a continuous variable.
Total diagnostic turnaround time	Defined as the time difference between sample collection and the date diagnostic test was ready (including sample collection, transportation, collection and diagnosis at the laboratory); it was also treated as a continuous variable.

a Initially, some of the returnees from abroad were tested for COVID-19 even in the absence of symptoms.

b For confirmed asymptomatic cases, period of contact was measured as the 2 days before, through the 14 days after the date on which the sample was taken which led to confirmation; for symptomatic cases, it was presumably 2 days before symptom onset through 14 days after.

c For more information on the criteria for urban/rural classification in Nigeria, see [12].

d All negative values following the subtraction of date variables were dropped.

### Data management and definition of study variables

De-identified data were retrieved from SORMAS. COVID-19 classifications (suspect, probable and confirmed case) were entered by trained data collectors as per the NCDC case definitions [10]. Data management and definitions of key study variables are presented in Table 1. The missing indicator approach was used to address missing data.

### Definition of outcome variables

The primary outcome variables for this study were cumulative incidence (CI) and case fatality (CF). CI was defined as the ratio of COVID-19 cases in a defined area to the estimated population of that area. Based on a national average growth rate of 3.2%, CI for each reporting state was calculated using the projected Nigerian population of 2020 from the 2006 national census and was multiplied by 100 000 for ease of interpretation. CF was defined as the proportion of persons diagnosed with COVID-19 who died during the study period, expressed as a percentage (%). Both CI and CF were calculated for Nigeria and for each state separately.

### Statistical analyses

Binary/categorical variables were described using frequencies and percentages (%), normally distributed continuous variables by means and standard deviations (s.d.), and non-normally distributed continuous variables by medians and interquartile ranges (IQR). Pearson *χ*^2^ test was used to assess how the sociodemographic and clinical characteristics between COVID-19 cases (confirmed cases *vs.* non-cases) and clinical outcome (alive *vs.* dead). A *P*-value of <0.05 was considered statistically significant. All statistical analyses were carried out in STATA version 13 (Stata Corp. LP, College Station, TX, United States of America). The report of this study was structured in accordance with the STROBE statement.

### Ethics

The study protocol was approved by the Nigeria National Health Research Ethics Committee (NHREC/01/01/2007-22/06/2020).

## Results

### Characteristics of the study population in relation to COVID-19 infection

**Fig. 1.**
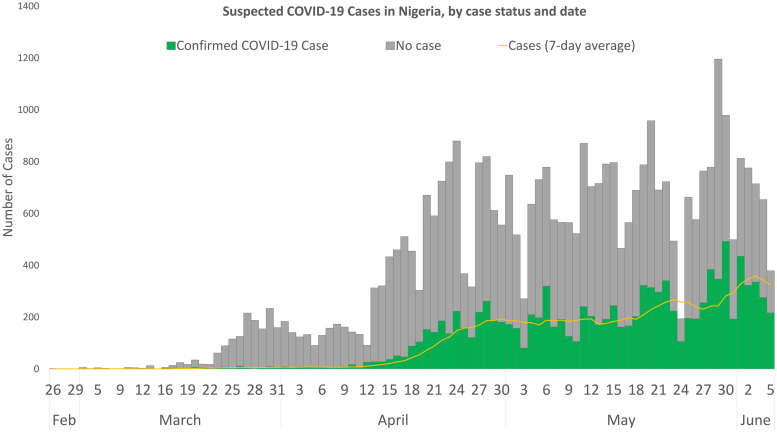
Suspected COVID-19 cases in Nigeria by case status and date, 26 February to 5 June 2020.

Between 27 February and 6 June 2020, 60 839 records were entered in the COVID-19 SORMAS database in Nigeria, these were classified as follows: 18 790 suspected cases (30.9%), 73 probable cases (0.1%), 12 289 confirmed cases (20.2%), 28 637 non-cases (47.1%) and 1050 non-classified cases (1.7%). This study focuses on individuals with definitive diagnostic classification (40 926): confirmed cases (*n* = 12 289) and non-cases (*n* = 28 637). The daily incidence of cases is shown in the epicurve in Figure 1.

**Fig. 2.**
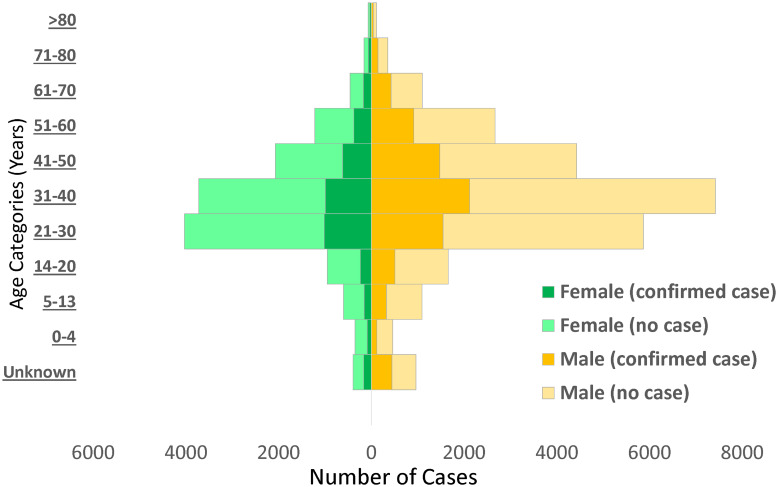
Distribution of non-cases and confirmed cases of COVID-19 by age and sex in Nigeria, 26 February to 6 June 2020.

Males (65.8%) constituted a higher proportion of confirmed COVID-19 cases than females (31.6%) (Fig. 2). The mean (s.d.) age of confirmed COVID-19 cases was 37.1 (15.7) years, with the highest proportion of these cases recorded among persons aged 31–40 years (25.5%) and 21–30 years (21.0%) (Table 2). Despite the high proportion of confirmed cases with missing information on education (53.1%), 30.6% reported completing tertiary education, followed by secondary school certificate holders at 8.6%. For confirmed cases with occupation information available, 9.3% were healthcare workers, while pupil/students and traders accounted for 6.7% each. The proportion of confirmed cases who reported history of travel 14 days prior to diagnosis was generally low, with local and international travels at 4.3% and 1.6%, respectively. Sixty-six per cent (8150/12 289) of confirmed COVID-19 cases were asymptomatic in the 14 days prior to diagnosis. Among confirmed COVID-19 cases with symptoms (*n* = 4139; 33.7%), fever (56.4%) and cough (55.5%) were the most common signs and symptoms reported. Other symptoms commonly reported among confirmed COVID-19 cases were runny nose (23.8%), sore throat (19.8%), difficulty in breathing (18.6%), headache (14.1%), diarrhoea (7.9%), nausea (7.5%), vomiting (5.5%) and fatigue (5.2%); other symptoms were less than 5.0% including loss of smell (4.1%) and loss of taste (3.1%).

**Table 2.** Distribution of sociodemographic and clinical characteristics of the study population in relation to COVID-19 infection status in Nigeria, 27 February–6 June 2020

Variable	Non-case (*n* = 28 637 (%)^a^)	Confirmed case (*n* = 12 289 (%))	Total case (*n* = 40 926 (%))
*Sociodemographic variables*			
Mean (s.d.) age, years^b^	35.0 (14.8)	37.1 (15.7)	35.6 (15.1)
Age group, years			
0–4	624 (2.2)	205 (1.7)	829 (2.0)
5–13	1225 (4.3)	482 (3.9)	1707 (4.2)
14–20	1885 (6.6)	752 (6.1)	2637 (6.4)
21–30	7397 (25.8)	2579 (21.0)	9976 (24.4)
31–40	8106 (28.3)	3128 (25.5)	11 234 (27.5)
41–50	4448 (15.5)	2114 (17.2)	6562 (16.0)
51–60	2634 (9.2)	1301 (10.6)	3935 (9.6)
61–70	963 (3.4)	602 (4.9)	1565 (3.8)
71–80	312 (1.1)	203 (1.7)	515 (1.3)
>80	114 (0.4)	71 (0.6)	185 (0.5)
Missing	929 (3.2)	852 (6.9)	1781 (4.4)^‡^
Sex			
Female	10 136 (35.4)	3880 (31.6)	14 016 (34.3)
Male	18 074 (63.1)	8086 (65.8)	26 160 (63.9)
Missing	427 (1.5)	323 (2.6)	750 (1.8)^‡^
Residential setting			
Rural	2664 (9.3)	719 (5.9)	3383 (8.3)
Urban	11 760 (41.1)	4786 (39.0)	16 546 (40.4)
Unknown	38 (0.1)	26 (0.2)	64 (0.2)
Missing	14 175 (49.5)	6758 (55.0)	20 933 (51.2)^‡^
Health facility			
Primary	2090 (7.3)	1087 (8.9)	3177 (7.8)
Secondary	1177 (4.1)	552 (4.5)	1729 (4.2)
Tertiary	1517 (5.3)	1206 (9.8)	2723 (6.7)
Missing	23 853 (83.3)	9444 (76.9)	33 297 (81.4)^‡^
Formal education completed			
None	563 (2.0)	194 (1.6)	757 (1.9)
Nursery/primary	936 (3.3)	276 (2.3)	1212 (3.0)
Secondary	2568 (9.0)	1057 (8.6)	3625 (8.9)
Tertiary	9719 (33.9)	3759 (30.6)	13 478 (32.9)
Alternative (e.g. Almajiranci)	730 (2.6)	482 (3.9)	1212 (3.0)
Missing	14 121 (49.3)	6521 (53.1)	20 642 (50.4)^‡^
Current occupation			
Pupil/student	2069 (7.2)	683 (5.6)	2752 (6.7)
Child	295 (1.0)	116 (0.9)	411 (1.0)
Housewife	382 (1.3)	150 (1.2)	532 (1.3)
Trader/business	1824 (6.4)	698 (5.7)	2522 (6.2)
Health worker	3544 (12.4)	1139 (9.3)	4683 (11.4)
Animal-related work	57 (0.2)	24 (0.2)	81 (0.2)
Farmer	468 (1.6)	111 (0.9)	579 (1.4)
Religious/traditional leader	66 (0.2)	32 (0.3)	98 (0.2)
Transporter	160 (0.6)	41 (0.3)	201 (0.5)
Other	11 860 (41.4)	4770 (38.8)	16 630 (40.6)
Missing	7912 (27.6)	4525 (36.8)	12 437 (30.4)^‡^
*Other epidemiological variables*			
Quarantine location			
Formal institution	591 (2.1)	458 (3.7)	1049 (2.6)
Informal institution (home)	3300 (11.5)	1167 (9.5)	4467 (10.9)
Missing	24 746 (86.4)	10 664 (86.8)	35 410 (86.5)^‡^
Travel history in last 14 days			
None	25 882 (90.4)	11 507 (93.6)	37 389 (91.4)
Local	1530 (5.3)	523 (4.3)	2053 (5.0)
International	1071 (3.7)	191 (1.6)	1262 (3.1)
Missing	154 (0.5)	68 (0.6)	222 (0.5)^‡^
*Clinical signs and symptoms among symptomatic persons only*^c^			
Mean (s.d.) temperature, °C	36.9 (1.1)	37.4 (1.0)	37.0 (1.1)
Abdominal pain			
No	8978 (96.5)	4000 (96.6)	12 978 (96.5)
Yes	103 (1.1)	31 (0.8)	134 (1.0)
Missing	226 (2.4)	108 (2.6)	334 (2.5)^†^
Chest pain			
No	8782 (94.4)	3876 (93.7)	12 658 (94.1)
Yes	309 (3.3)	160 (3.8)	469 (3.5)
Missing	216 (2.3)	103 (2.5)	319 (2.4)^‡^
Chills/sweat			
No	8981 (96.5)	3973 (96.0)	12 954 (96.3)
Yes	104 (1.1)	63 (1.5)	167 (1.2)
Missing	222 (2.4)	103 (2.5)	325 (2.4)^‡^
Confusion			
No	9036 (97.1)	3990 (96.4)	13 026 (96.9)
Yes	29 (0.3)	14 (0.3)	43 (0.3)
Missing	242 (2.6)	135 (3.3)	377 (2.8)^‡^
Cough			
No	4355 (46.8)	1816 (43.9)	6171 (45.9)
Yes	4865 (52.3)	2298 (55.5)	7163 (53.3)
Missing	87 (0.9)	25 (0.6)	112 (0.8)^‡^
Diarrhoea			
No	8316 (89.4)	3731 (90.1)	12 047 (89.6)
Yes	815 (8.8)	327 (7.9)	1142 (8.5)
Missing	176 (1.9)	81 (2.0)	257 (1.9)^‡^
Difficulty in breathing			
No	7231 (77.7)	3280 (79.3)	10 511 (78.2)
Yes	1892 (20.3)	768 (18.6)	2660 (19.8)
Missing	184 (2.0)	91 (2.2)	275 (2.1)^‡^
Fatigue			
No	8792 (94.5)	3816 (92.2)	12 608 (93.8)
Yes	294 (3.2)	216 (5.2)	510 (3.8)
Missing	221 (2.4)	107 (2.6)	328 (2.4)^‡^
Fever			
No	5314 (57.1)	1775 (42.9)	7089 (52.7)
Yes	3941 (42.3)	2334 (56.4)	6275 (46.7)
Missing	52 (0.6)	30 (0.7)	82 (0.6)^‡^
Headache			
No	8283 (89.0)	3475 (84.0)	11 758 (87.5)
Yes	808 (8.7)	583 (14.1)	1391 (10.4)
Missing	216 (2.3)	81 (1.9)	297 (2.2)^‡^
Joint pain			
No	8975 (96.4)	3975 (96.0)	12 950 (96.3)
Yes	104 (1.1)	54 (1.3)	158 (1.2)
Missing	228 (2.5)	110 (2.7)	338 (2.5)^‡^
Malaise			
No	5588 (60.0)	1920 (46.4)	7508 (55.8)
Yes	100 (1.1)	92 (2.2)	192 (1.4)
Missing	3619 (38.9)	2127 (51.4)	5746 (42.7)^‡^
Muscle pain			
No	8842 (95.0)	3936 (95.1)	12 778 (95.0)
Yes	231 (2.5)	96 (2.3)	327 (2.4)
Missing	234 (2.5)	107 (2.6)	341 (2.5)^‡^
Nausea			
No	8393 (90.2)	3736 (90.3)	12 129 (90.2)
Yes	729 (7.8)	311 (7.5)	1040 (7.7)
Missing	185 (2.0)	92 (2.2)	277 (2.1)^‡^
Pharyngeal exudate			
No	5638 (60.6)	1996 (48.2)	7634 (56.8)
Yes	9 (0.1)	3 (0.1)	12 (0.1)
Missing	3660 (39.3)	2140 (51.7)	5800 (43.1)^‡^
Rapid breathing			
No	7467 (80.2)	2932 (70.8)	10 399 (77.3)
Yes	115 (1.2)	61 (1.5)	176 (1.3)
Missing	1725 (18.5)	1146 (27.7)	2871 (21.4)^‡^
Runny nose			
No	6776 (72.8)	3067 (74.1)	9843 (73.2)
Yes	2377 (25.5)	986 (23.8)	3363 (25.0)
Missing	154 (1.7)	86 (2.1)	240 (1.8)^‡^
Sore throat			
No	6562 (70.5)	3276 (79.1)	9838 (73.2)
Yes	2590 (27.8)	818 (19.8)	3408 (25.3)
Missing	155 (1.7)	45 (1.1)	200 (1.5)^‡^
Vomiting			
No	8552 (91.9)	3823 (92.4)	12 375 (92.0)
Yes	577 (6.2)	228 (5.5)	805 (6.0)
Missing	178 (1.9)	88 (2.1)	266 (2.0)^‡^
Loss of smell			
No	5204 (55.9)	2926 (70.7)	8130 (60.4)
Yes	43 (0.5)	170 (4.1)	213 (1.6)
Missing	4060 (43.6)	1043 (25.2)	5103 (38.0)^‡^
Loss of taste			
No	5200 (55.9)	2966 (71.7)	8166 (60.7)
Yes	49 (0.5)	130 (3.1)	179 (1.3)
Missing	4058 (43.6)	1043 (25.2)	5101 (38.0)^‡^
Conjunctival injection			
No	9058 (97.3)	4017 (97.1)	13 075 (97.2)
Yes	17 (0.2)	13 (0.3)	30 (0.2)
Missing	232 (2.5)	109 (2.6)	341 (2.5)^‡^

a Percentages in some instances may be greater than 100.0% due to rounding up.

b Based on 39 145 observations.

c Analyses were restricted to individuals who showed symptoms during the study period: non-cases (9307), confirmed cases (4139) and total cases (13 446).

† *P*-value <0.05.

‡ *P*-value <0.001.

### Characteristics of the study population in relation to clinical outcome among confirmed COVID-19 cases

Overall, 3467 out of 12 289 confirmed COVID-19 cases had complete records on clinical outcome over the period covered by this study: 3125 surviving *vs.* 342 dead (Table 3). Mean age of persons who died from COVID-19 was 55.5 (16.4) years. Overall, death from COVID-19 infection increased with increasing age, reaching its highest proportion at 26.6% among persons aged 61–70 years. More deaths were among males (79.0%) than females (19.9%), similar to the gender distribution of COVID-19 cases. Regarding the occupation of those who died, 7.3% of death was recorded among traders, while 40.6% had missing data. Among those who died from COVID-19, 62.0% (212/342) had shown at least one clinical symptom in the 14 days prior to diagnosis (results not shown in Table 3). Of these, cough (72.6%), fever (64.6%) and difficulty in breathing (51.4%) were the most commonly recorded signs and symptoms. Other common symptoms recorded at diagnosis were sore throat (16.5%), runny nose (15.1) and vomiting (12.3%).

**Table 3.** Distribution of sociodemographic and clinical characteristics of the study population in relation to clinical outcome from COVID-19 infection

Variable	Clinical outcome among COVID-19 cases^a^		
	Survivor (*n* = 3125 (%^b^))	Dead (*n* = 342 (%))	Total (*n* = 3467 (%))
*Sociodemographic characteristics*			
Mean (s.d.) age, years	35.6 (15.3)	55.5 (16.4)	37.1 (15.7)^Φ^
Age group, years			
0–4	32 (1.0)	1 (0.3)	33 (0.9)
5–13	185 (5.9)	1 (0.3)	186 (5.4)
14–20	291 (9.3)	6 (1.8)	297 (8.6)
21–30	646 (20.7)	24 (7.0)	670 (19.3)
31–40	809 (25.9)	30 (8.8)	839 (24.2)
41–50	534 (17.1)	47 (13.7)	581 (16.8)
51–60	346 (11.1)	81 (23.7)	427 (12.3)
61–70	121 (3.9)	91 (26.6)	212 (6.1)
71–80	37 (1.2)	36 (10.5)	73 (2.1)
>80	12 (0.4)	11 (3.2)	23 (0.7)
Missing	112 (3.6)	14 (4.1)	126 (3.6)‡
Sex			
Female	925 (29.6)	68 (19.9)	993 (28.6)
Male	2188 (70.0)	270 (79.0)	2458 (70.9)
Missing	12 (0.4)	4 (1.1)	16 (0.5)‡
Residential setting			
Rural	244 (7.8)	22 (6.4)	266 (7.7)
Urban	1357 (43.4)	157 (45.9)	1514 (43.7)
Unknown	4 (0.1)	0 (0.0)	4 (0.1)
Missing	1520 (48.6)	163 (47.7)	1683 (48.5)NS
Health facility			
Primary	275 (8.8)	5 (1.5)	280 (8.1)
Secondary	186 (6.0)	22 (6.4)	208 (6.0)
Tertiary	274 (8.8)	43 (12.6)	317 (9.1)
Missing	2390 (76.5)	272 (79.5)	2662 (76.8)‡
Formal education completed			
None	124 (4.0)	8 (2.3)	132 (3.8)
Nursery/primary	94 (3.0)	6 (1.8)	100 (2.9)
Secondary	306 (9.8)	30 (8.8)	336 (9.7)
Tertiary	1072 (34.3)	102 (29.8)	1174 (33.9)
Alternative (e.g. Almajiranci)	100 (3.2)	11 (3.2)	111 (3.2)
Missing	1429 (45.7)	185 (54.1)	1614 (46.6)NS
Current occupation			
Pupil/student	209 (6.7)	3 (0.9)	212 (6.1)
Child	38 (1.2)	0 (0.0)	38 (1.1)
Housewife	59 (1.9)	8 (2.3)	67 (1.9)
Trader/business	212 (6.8)	25 (7.3)	237 (6.8)
Health worker	343 (11.0)	12 (3.5)	355 (10.2)
Animal-related work	6 (0.2)	0 (0.0)	6 (0.2)
Farmer	40 (1.3)	14 (4.1)	54 (1.6)
Religious/traditional leader	12 (0.4)	4 (1.2)	16 (0.5)
Transporter	12 (0.4)	2 (0.6)	14 (0.4)
Other	1193 (38.2)	135 (39.5)	1328 (38.9)
Missing	1001 (32.0)	139 (40.6)	1140 (32.9)‡
*Other epidemiological variables*			
Quarantine location			
Formal institution	299 (9.6)	27 (7.9)	326 (9.4)
Home	464 (14.9)	48 (14.0)	512 (14.8)
Missing	2362 (75.6)	267 (78.1)	2629 (75.8)NS
Travel history in last 14 days			
None	2696 (86.3)	317 (92.7)	3013 (86.9)
Local	284 (9.1)	16 (4.7)	300 (8.7)
International	108 (3.5)	4 (1.2)	112 (3.2)
Missing	37 (1.2)	5 (1.5)	42 (1.2)‡
*Clinical signs and symptoms among symptomatic persons only*^c^			
Mean (s.d.) temperature, °C^d^	37.4 (1.0)	37.5 (1.3)	37.4 (1.1)
Abdominal pain			
No	1286 (94.1)	198 (93.4)	1484 (94.0)
Yes	14 (1.0)	6 (2.8)	20 (1.3)
Missing	66 (4.8)	8 (3.8)	74 (4.7)‡
Chest pain			
No	1251 (91.6)	191 (90.1)	1442 (91.4)
Yes	53 (3.9)	14 (6.6)	67 (4.3)
Missing	62 (4.5)	7 (3.3)	69 (3.4)‡
Chills/sweat			
No	1281 (93.8)	201 (94.8)	1482 (93.9)
Yes	26 (1.9)	3 (1.4)	29 (1.8)
Missing	59 (4.3)	8 (3.8)	67 (4.3)‡
Confusion			
No	1298 (95.0)	200 (94.3)	1498 (94.9)
Yes	4 (0.3)	4 (1.9)	8 (0.5)
Missing	64 (4.7)	8 (3.8)	72 (4.6)‡
Cough			
No	607 (44.4)	55 (25.9)	662 (42.0)
Yes	751 (55.0)	154 (72.6)	905 (57.3)
Missing	8 (0.6)	3 (1.4)	11 (0.7)‡
Diarrhoea			
No	1206 (88.3)	186 (87.7)	1392 (88.2)
Yes	112 (8.2)	20 (9.4)	132 (8.4)
Missing	48 (3.5)	6 (2.8)	54 (3.4)‡
Difficulty in breathing			
No	1066 (78.0)	98 (46.2)	1164 (73.8)
Yes	237 (17.4)	109 (51.4)	346 (21.9)
Missing	63 (4.6)	5 (2.4)	68 (4.3)‡
Fatigue			
No	1207 (88.4)	182 (85.9)	1389 (88.0)
Yes	93 (6.8)	24 (11.3)	117 (7.4)
Missing	66 (4.8)	6 (2.8)	72 (4.6)‡
Fever			
No	498 (36.4)	73 (34.4)	571 (36.1)
Yes	855 (62.6)	137 (64.6)	992 (62.9)
Missing	13 (1.0)	2 (1.0)	15 (1.0)‡
Headache			
No	1070 (78.3)	186 (87.7)	1256 (79.6)
Yes	251 (18.4)	20 (9.4)	271 (17.2)
Missing	45 (3.3)	6 (2.8)	51 (3.2)‡
Joint pain			
No	1278 (93.6)	199 (93.9)	1477 (93.6)
Yes	22 (1.6)	6 (2.8)	28 (1.8)
Missing	66 (4.8)	7 (3.3)	73 (4.6)‡
Malaise			
No	957 (70.1)	102 (48.1)	1059 (67.1)
Yes	60 (4.4)	13 (6.1)	73 (4.6)
Missing	349 (25.6)	97 (45.8)	446 (28.3)‡
Muscle pain			
No	1253 (91.7)	199 (93.9)	1452 (92.0)
Yes	49 (3.6)	6 (2.8)	55 (3.5)
Missing	64 (4.7)	7 (3.3)	71 (4.5)‡
Nausea			
No	1219 (89.2)	189 (89.2)	1408 (89.2)
Yes	87 (6.4)	16 (7.6)	103 (6.5)
Missing	60 (4.4)	7 (3.3)	67 (4.3)‡
Rapid breathing			
No	1040 (76.1)	137 (64.6)	1177 (74.6)
Yes	21 (1.5)	11 (5.2)	32 (2.0)
Missing	305 (22.3)	64 (30.2)	369 (23.4)‡
Runny nose			
No	1011 (74.0)	174 (82.1)	1185 (75.1)
Yes	297 (21.7)	32 (15.1)	329 (20.9)
Missing	58 (4.3)	6 (2.8)	64 (4.1)‡
Sore throat			
No	1052 (77.0)	175 (82.6)	1227 (77.8)
Yes	296 (21.7)	35 (16.5)	331 (21.0)
Missing	18 (1.3)	2 (0.9)	20 (1.2)‡
Vomiting			
No	1247 (91.3)	181 (85.4)	1428 (90.5)
Yes	59 (4.3)	26 (12.3)	85 (5.4)
Missing	60 (4.4)	5 (2.4)	65 (4.1)‡
Loss of smell			
No	762 (55.8)	135 (63.7)	897 (56.8)
Yes	22 (1.6)	1 (0.5)	23 (1.5)
Missing	582 (42.6)	76 (35.9)	658 (41.7)NS
Loss of taste			
No	760 (55.6)	132 (62.3)	892 (56.5)
Yes	25 (1.8)	5 (2.4)	30 (1.9)
Missing	581 (42.5)	75 (35.3)	656 (41.6)NS
Conjunctival injection			
No	1294 (94.7)	205 (96.7)	1499 (95.0)
Yes	6 (0.4)	0 (0.0)	6 (0.4)
Missing	66 (4.8)	7 (3.3)	73 (4.6)‡
Pneumonia			
No	1289 (94.3)	201 (94.8)	1.490 (94.4)
Yes	1 (0.1)	3 (1.4)	4 (0.3)
Missing	76 (5.6)	8 (3.8)	84 (5.3)‡
ARDS			
No	1287 (94.2)	196 (92.5)	1483 (94.0)
Yes	16 (1.2)	10 (4.7)	26 (1.6)
Missing	63 (4.6)	6 (2.8)	69 (4.4)‡

ARDS, acute respiratory distress syndrome.

a 8822 persons diagnosed with COVID-19 did not yet have a clinical outcome during the study period.

b Percentages in some instances may be greater than 100.0% due to rounding up.

c Only for symptomatic confirmed COVID-19 cases with records of clinical outcome: survivor (*n* = 1366), dead (*n* = 212), and total (*n* = 1578).

d 692 total records were used for the assessment of temperature.

†*P*-value <0.05; ‡ *P*-value <0.001; NS = *P*-value not statistically significant (i.e. >0.05).

Φ: *P*-value from *t*-test was <0.0001; mean difference was 19.9 years.

### Description of clinical time variables

Table 4 describes the timelines for clinical variables from available records during this study period. Based on the records of 8370 confirmed COVID-19 cases, the median (IQR) turnaround time for laboratory diagnosis was 2 (1–4) days, whereas it was 1 (1–3) day among 17 817 non-cases. Median (IQR) time from self-reported onset of symptom to sample collection for laboratory diagnosis among 2426 confirmed COVID-19 cases with symptom presentation was 7 (2–17) days. Among 186 deaths from COVID-19 infection, the median (IQR) time from sample collection for laboratory diagnosis to death was 4 (1–8) days.

**Table 4.** Description of time of available clinical variables among COVID-19 cases

Time variable	Confirmed case		Non-case	
	Total cases with data (*N*)	Median number (IQR) of days	Total cases with data (*N*)	Median number (IQR) of days
Time from symptom onset to sample collection for diagnosis	2426	7 (2–17)	5481	5 (1–12)
Duration in quarantine	259	14 (12–42)	808	14 (2–43)
Duration on admission in hospital	111	19 (9–41)	34	7 (1–61)
Time from sample collection to death	186	4 (1–8)	39	1 (0–28)
Time from sample collection to transportation/shipment to the laboratory	2541	0 (0–2)	7847	0 (0–1)
Total laboratory turnaround time	8370	2 (1–4)	17 817	1 (1–3)

### Cumulative incidence of COVID-19 and case fatality in Nigeria, 27 February–6 June 2020

**Fig. 3.**
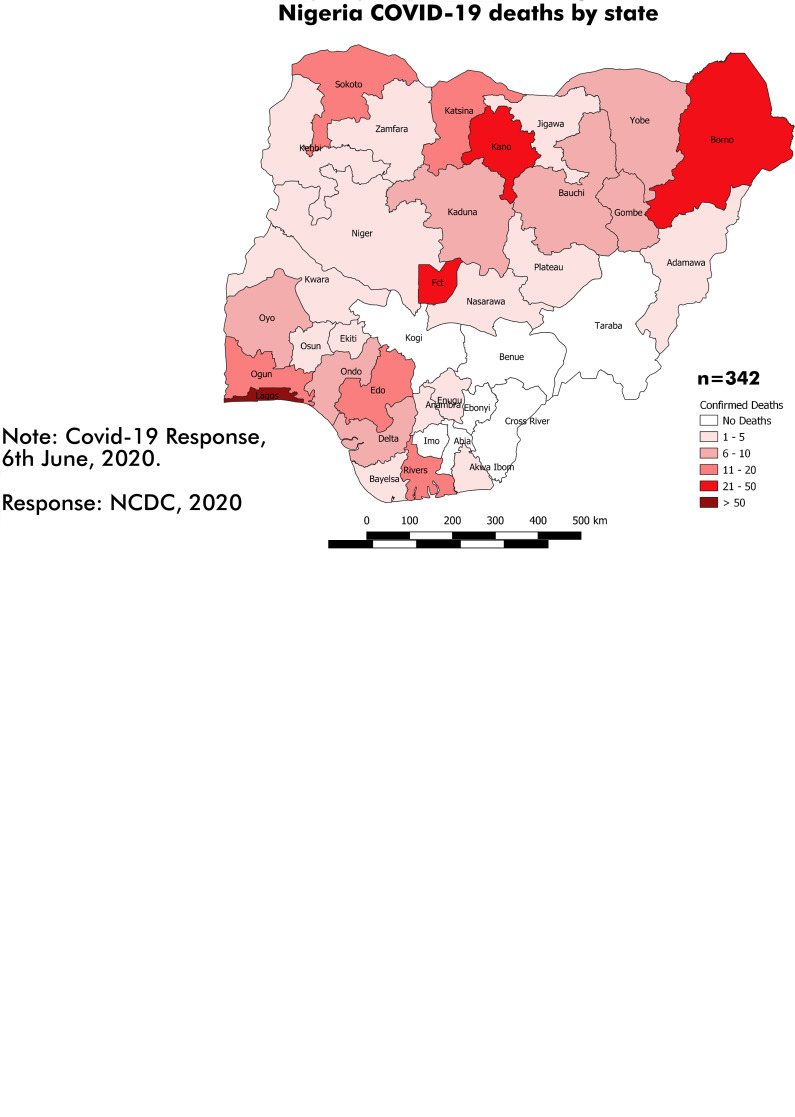
(a) Spatial distribution of confirmed COVID-19 cases by Nigerian State, 28 February–6 June 2020. (b) Spatial distribution of confirmed COVID-19 deaths by Nigerian State, 28 February–6 June 2020.

The overall CI of COVID-19 infection and CF in Nigeria during the study period was 5.6 per 100 000 population and 2.8%, respectively (Table 5). Lagos State (39.9 per 100 000), followed by the FCT (19.4 per 100 000), recorded the highest CI in Nigeria during this study period. Other States with CI higher than the national figure include Edo (8.6 per 100 000), Kano (6.8 per 100 000), Ogun (5.9 per 100 000) and Gombe (5.7 per 100 000). Regarding CF across the various Nigerian States, Ondo recorded the highest figure at 16.7%, followed by Yobe State (13.5%), Kebbi State (11.4%) and Bayelsa State (10.0%). The spatial distribution of confirmed COVID-19 cases and death by state is presented in Figure 3.

**Table 5.** CI of COVID-19 and CF rate in Nigeria, 27 February–6 June 2020

Variable	Population at risk	Number of confirmed COVID-19 cases	CI/100 000 population	Number of deaths from COVID-19	CF rate (%)
*State*^a^					
Lagos	14 009 120	5587	39.9	72	1.3
FCT	4 880 010	945	19.4	23	2.4
Edo	4 673 229	403	8.6	19	4.7
Kano	14 783 518	999	6.8	47	4.7
Ogun	5 873 430	349	5.9	12	3.4
Gombe	3 658 473	207	5.7	10	4.8
Borno	6 629 190	350	5.3	22	6.3
Oyo	8 929 410	422	4.7	7	1.7
Katsina	8 761 794	397	4.5	20	5.0
Rivers	8 280 753	362	4.4	18	5.0
Jigawa	6 488 878	283	4.4	7	2.5
Ebonyi	3 199 362	135	4.2	0	0.0
Kwara	3 586 485	142	4.0	1	0.7
Kaduna	9 176 219	344	3.7	11	3.2
Bauchi	7 468 016	273	3.7	2	0.7
Nasarawa	2 818 371	78	2.8	4	5.1
Plateau	4 615 698	119	2.6	2	1.7
Delta	6 369 849	155	2.4	8	5.2
Sokoto	5 592 043	129	2.3	14	10.9
Zamfara	5 066 556	77	1.5	5	6.5
Yobe	3 757 947	52	1.4	7	13.5
Bayelsa	2 541 682	30	1.2	3	10.0
Osun	5 320 967	49	0.9	4	8.2
Adamawa	4 727 312	42	0.9	4	9.5
Ondo	5 204 858	42	0.8	7	16.7
Abia	4 115 152	31	0.8	0	0.0
Ekiti	3 655 663	29	0.8	2	6.9
Niger	6 308 295	45	0.7	1	2.2
Akwa-Ibom	6 260 322	45	0.7	2	4.4
Kebbi	4 965 722	35	0.7	4	11.4
Imo	6 115 745	36	0.6	0	0.0
Enugu	4 926 955	29	0.6	1	3.4
Taraba	3 402 844	19	0.6	0	0.0
Anambra	6 155 892	32	0.5	3	9.4
Benue	6 381 985	15	0.2	0	0.0
Kogi^b^	4 959 006	2	0.0	0	0.0
Overall^c^	217 971 548	12 289	5.6	342	2.8

a Excluding Cross River State with no official report of COVID-19 during the study period.

b 0.04 when values were not rounded to one decimal place.

c Based on the 2020 projected Nigerian population.

## Discussion

### Summary of key findings

We have provided a description of the first national epidemiology of COVID-19 cases and associated clinical features and outcomes for Nigeria. There were 12 289 confirmed COVID-19 cases and 28 637 non-cases in 35 states plus the FCT in Nigeria between 27 February and 6 June 2020. During this period, there were 342 deaths, a CI of 5.6 per 100 000 and a CF of 2.8% overall.

### Interpretation of key findings

After South Africa, Nigeria is the second most-affected African country in terms of recorded confirmed COVID-19 cases and death as of 7 June 2020 [14]. However, the CI of COVID-19 in Nigeria during the study period, at 5.6 per 100 000 population, is substantially lower than in some non-African countries at a similar stage in their epidemic. For example, about three months after the first confirmed case in the United States, CI was 119⋅6 per 100 000 population, far more than that of Nigeria's; with Minnesota, the State with the lowest CI, having a CI of 20.6 per 100 000 population [15]. Additionally, many European countries reached a CI of at least 4.0 confirmed cases per 100 000 population over a period of less than 1 month [16]. A possible reason for lower CI in Nigeria could be due to a relatively low testing capacity in the country as compared to the US and European countries.

There was substantial variability in COVID-19 incidence among the states in Nigeria. The heterogeneity in CIs within Nigeria could be attributable, in part, to international travels as indicated by the figures recorded by Lagos State (39.9 per 100 000) and the FCT (19.4 per 100 000) with the two major international airports in the country. Another possible explanation might be due to variations in the estimated population of states in Nigeria, with smaller population recording a higher CI and vice-versa. For example, Ekiti State (3 655 663 population) and Enugu State (4 926 955 population) each recorded 29 confirmed COVID-19 cases during this study period; but the latter recorded a lower CI (0.6 per 100 000 population) than the former (0.8 per 100 000). Moreover, all the Nigerian states did not have a similar testing capacity during the study period, and this might have contributed to the observed findings in terms of the numerator figures for calculating CIs. Similarly, the CF of 2.8% in this study is lower than several other countries which have been hard hit by the COVID-19 pandemic. There is a wide range of CFs among non-African countries (from 0.1% in Singapore to 16.2% in Belgium [17]) and in African countries (from 0.0% in Uganda to 8.2% in Chad) during this study period [14]. Nigeria's observed CF of 2.8% is on the lower end of the range reported outside and within Africa, but higher than the 2.4% (3210 deaths/133 119 confirmed cases) recorded for the entire Africa as of 7 June 2020 [14]. The variation in CF in Nigeria could be an indication of varying health system capacity and preparedness across the country. An unpublished study indicates that Lagos State – with the highest CI but a CF of 1.3% – invested substantially in case management of COVID-19 patients as part of its preparedness activities. The overall CF in Nigeria could be partly due to its much younger population compared to the United States and most countries in Europe [18]; similar trends in deaths by age from COVID-19 have been reported in China [19]. Just as cases are potentially underestimated due to inadequate testing, it is likely that deaths from COVID-19 are also underestimated, especially in places like Kano, which reported significant increases in deaths in April [20].

In contrast to deaths from COVID-19, a higher proportion of COVID-19 cases was recorded among economically active age groups, suggesting potential role of socio-economic or work-related activities rather than immunological capacity. Children under 5 years of age and those aged 5–13 years, respectively, accounted for 1.7% and 3.9% of confirmed COVID-19 cases in this study. These findings are comparable to those from a recent global systematic review [21]. Although it remains unclear why children are less affected by COVID-19 than older individuals, evidence suggests differences in immune system function [22]. The higher infection rate among males in this study corresponds to evidence reported in the WHO African region, where males in the 31–39 and 40–49 age groups accounted for 62% of 5178 recorded cases [14]. Outside Africa, early findings of the clinical characteristics of 41 confirmed COVID-19 cases in Wuhan, China, reported males to have accounted for 30 (73.0%) of the cases [23]. A study in Italy also reported male preponderance [24]. A combination of genetic and physiological factors has been hypothesised as possible explanations for the potential male bias. For example, the wider distribution of SARS-CoV-2 cellular receptor, angiotensin-converting enzyme 2 (ACE-2), in male over females has been postulated [25]. In a patriarchal system such as seen in Nigeria, men are more likely to engage in economic activities outside of the household and potentially become more exposed to SARS-CoV-2 infection than women. While this may be more feasible during a controlled economy, such as that seen during the suspension of non-essential economic activities in the early phase of COVID-19 outbreak in Nigeria, it may not be applicable when socio-economic activities are functional. This is because women are increasingly partaking in the workforce in Nigeria, such that the traditional trends of ‘male breadwinner and female family support’ are fast eroding [26].

The median length of stay of 111 patients with COVID-19 in hospital in this study was 19 days, which is within the range outside of China (4–21 days), but comparatively lower than that from China (4–53 days) [27]. In general, differences in the length of hospital stay may be attributable to variations in criteria for admission and discharge across different countries as well as timing within the pandemic [27]. Early diagnosis is fundamental for effective management of COVID-19 cases; thus, a median turnaround time of 2 (1–4) days for laboratory diagnosis as noted in the current study seems impressive, and possibly an indication of ongoing measures being championed by the NCDC to strengthen molecular diagnostic capacity in Nigeria. However, we lacked information on when laboratory test was received by a COVID-19 suspected case, as turnaround time only included the time from sample collection to availability of result.

The symptomatic status of confirmed COVID-19 cases in this analysis is noteworthy, as over half of them were asymptomatic at testing. A scoping review of the literature found that between 5% and 80% of people testing positive for SARS-CoV-2 may be asymptomatic [28], placing the 66% in the current study closer to the maximum range. It is possible that the case investigation approach adopted during testing might have underestimated symptoms: patients were initially asked whether they were symptomatic and probed about individual symptoms only if they answered in the affirmative. Stigma associated with COVID-19 in Nigeria might contribute to people not reporting symptoms when they get tested [29]. Furthermore, it is possible for asymptomatic status at diagnosis to change in the course of an illness, in which case such persons could be better classified as pre-symptomatic cases, so the proportion of truly asymptomatic cases cannot be described by these data. Nevertheless, this scenario could pose a challenge to community surveillance activities and implementation of public health interventions (e.g. quarantine and isolation). Thus, the possibility of COVID-19 transmission by asymptomatic cases in Nigeria needs to be explored and addressed, both in terms of research and community risk communication activities.

The most common signs and symptoms among symptomatic confirmed COVID-19 cases in the 14 days prior to diagnosis were fever (56.4%) and cough (55.5%). This trend is similar to that recorded in a recent systematic review of the literature for China [30]; however, while fatigue was the third most frequently recorded symptom in China, its frequency was low in our study at 5.2%. Similarly, cough, fever and difficulty in breathing, in that order, were the most commonly recorded symptoms at diagnosis among persons who died from COVID-19 infection. The common occurrence of difficulty in breathing in deceased patients has been identified as a major driver of adverse clinical outcomes among COVID-19 patients [31]. Although relatively small in proportion due to late recording during the study period, loss of smell and loss of taste among confirmed COVID-19 cases in this study are consistent with available evidence [32]. However, being a descriptive study, these data do not have the capacity to establish a causal association between observed clinical symptoms and COVID-19 infection or death. Thus, a follow-up study aimed at exploring these associations is recommended. It is also worth noting that the fever which is one of the common symptoms noted in this study is often common in endemic febrile illnesses in Nigeria including malaria, Lassa fever and yellow fever. As such, in the case of a co-infection, misclassification of illnesses is likely if symptoms alone are used for COVID-19 case definitions [33]. The symptomatic and geographic convergence of COVID-19 and common febrile diseases in Nigeria therefore requires continuous strengthening of definitive diagnostic approaches in the country. About 9% of COVID-19 infections occurred in healthcare workers during this study period. COVID-19 infection among health workers is of prominent public health importance as it could potentially enhance disease transmission [34] and further weaken a health system that already struggles with insufficient human resources for health.

### Strengths and limitations

This study has provided the first national epidemiological evidence on COVID-19 in Nigeria, necessary for public health planning and health system strengthening. However, this study is limited by the substantial proportion of missing data within some of the sociodemographic (e.g. residential setting and health facility) and clinical (e.g. malaise, pharyngeal exudate, rapid breathing, loss of smell and taste) variables studied. The late addition of loss of smell and taste to the CIF in Nigeria may partly explain why data recorders were not accustomed to capturing them. The high proportion of missing data on some key indicators has prompted a systemic effort to improve the quality of SORMAS data, and a dedicated Data Quality Improvement Project (DQIP) was initiated in April to improve completeness of key variables to above 90%.

In conclusion, this study has provided an early insight into the epidemiology of COVID-19 in Nigeria. Evidence from this study, such as the high proportion of cases among the active age group and high proportion of asymptomatic cases at diagnosis, will be useful for policymakers and stakeholders in the health and other sectors in contextualising public health planning and response as well as for scientific activities in the country. Such measures could include intensifying NPIs at work and commercial places where this age group is mostly found, and adapting case finding protocols to include routine testing of asymptomatic contacts of confirmed cases.

## Data Availability

Readers can contact the corresponding author at chinwe.ochu@ncdc.gov.ng for access to the data used for this study.

## References

[1] Gralinski LE and Menachery VD (2020) Return of the coronavirus: 2019-nCoV. Viruses 12. doi: 10.3390/v12020135. PMC7077245 31991541

[2] World Health Organization (2020) Pneumonia of unknown cause-China. WHO.

[3] Sohrabi C (2020) World Health Organization declares global emergency: a review of the 2019 novel coronavirus (COVID-19). International Journal of Surgery 76, 71–76. 32112977 10.1016/j.ijsu.2020.02.034 PMC7105032

[4] Zhao S (2020) Preliminary estimation of the basic reproduction number of novel coronavirus (2019-nCoV) in China, from 2019 to 2020: a data-driven analysis in the early phase of the outbreak. International Journal of Infectious Diseases 92, 214–217. 32007643 10.1016/j.ijid.2020.01.050 PMC7110798

[5] Nigeria Centre for Disease Control (2020) COVID-19 outbreak in Nigeria: situation report-009. pp. 1–3.

[6] Ebenso B and Otu A (2020) Can Nigeria contain the COVID-19 outbreak using lessons from recent epidemics? The Lancet Global Health 8, e770. 32171055 10.1016/S2214-109X(20)30101-7 PMC7104043

[7] Bowale A (2020) Clinical presentation, case management and outcomes for the first 32 COVID-19 patients in Nigeria. Pan African Medical Journal 35, 24–27. 33623549 10.11604/pamj.supp.2020.35.2.23262 PMC7875732

[8] Nigeria Centre for Disease Control (2020) An update of COVID-19 outbreak in Nigeria. https://ncdc.gov.ng

[9] Fall IS (2019) Integrated Disease Surveillance and Response (IDSR) strategy: current status, challenges and perspectives for the future in Africa. BMJ Global Health 4, e001427. 10.1136/bmjgh-2019-001427 PMC6615866 31354972

[10] Nigeria Centre for Disease Control (2020) National Interim Guidelines for Clinical Management of COVID-19, Version 2, May 2020. Abuja.

[11] World Health Organization (2020) Laboratory testing for coronavirus disease (COVID-19) in suspected human cases: Interim guidance, 19 March 2020. WHO.

[12] Ofem BI (2012) A review of the criteria for defining urban areas in Nigeria. Journal of Human Ecology 37, 167–171.

[13] Federal Ministry of Health (2020) Nigeria Health Facility Registry. *Nigeria Health Facility Registry*.

[14] World Health Organization Africa (2020) Weekly Bulletin on Outbreaks and Other Emergencies: Week 23: 1–7 June 2020.

[15] CDC COVID-19 Response Team (2020) Geographic differences in COVID-19 cases, deaths, and incidence – United States, February 12–April 7, 2020. Morbidity and Mortality Weekly Report US 69, 465–471. 10.15585/mmwr.mm6915e4 PMC7755058 32298250

[16] Kinross P (2020) Rapidly increasing cumulative incidence of coronavirus disease (COVID-19) in the European Union/European Economic Area and the United Kingdom, 1 January to 15 March 2020. Eurosurveillance 25, 2000285. 10.2807/1560-7917.ES.2020.25.11.2000285 PMC7096777 32186277

[17] Johns Hopkins Coronavirus Resource Center (2020) Mortality analyses. Maps and Trends. https://coronavirus.jhu.edu/data/mortality

[18] Dowd JB (2020) Demographic science aids in understanding the spread and fatality rates of COVID-19. Proceedings of the National Academy of Sciences of the United States of America 117, 9696–9698. 32300018 10.1073/pnas.2004911117 PMC7211934

[19] Liu K (2020) Clinical features of COVID-19 in elderly patients: a comparison with young and middle-aged patients. Journal of Infection 80, e14–e18. 10.1016/j.jinf.2020.03.005 PMC7102640 32171866

[20] Centre for Democracy & Development (2020) Unpacking Falsehoods: COVID-19 and Responses in Kano State.

[21] Ludvigsson JF (2020) Children are unlikely to be the main drivers of the COVID-19 pandemic – a systematic review. *Acta Paediatrica*. International Journal of Paediatrics 109, 1525–1530. 10.1111/apa.15371 PMC7280674 32430964

[22] Balasubramanian S (2020) Coronavirus disease (COVID-19) in children – what we know so far and what we do not? Indian Pediatrics 57, 435–442. 32273490 10.1007/s13312-020-1819-5 PMC7240240

[23] Huang C (2020) Clinical features of patients infected with 2019 novel coronavirus in Wuhan, China. The Lancet 395, 497–506. 10.1016/S0140-6736(20)30183-5 PMC7159299 31986264

[24] Grasselli G (2020) Baseline characteristics and outcomes of 1591 patients infected with SARS-CoV-2 admitted to ICUs of the Lombardy region, Italy. Journal of the American Medical Association 323, 1574–1581. 32250385 10.1001/jama.2020.5394 PMC7136855

[25] Patel SK (2013) Emerging markers in cardiovascular disease: where does angiotensin-converting enzyme 2 fit in? Clinical and Experimental Pharmacology and Physiology 40, 551–559. 23432153 10.1111/1440-1681.12069

[26] Akanle O (2018) Turbulent but I must endure in silence: female breadwinners and survival in southwestern Nigeria. Journal of Asian and African Studies 53, 98–114.

[27] Rees EM (2020) COVID-19 length of hospital stay: a systematic review and data synthesis. medRxiv 18, 270–291. 10.1186/s12916-020-01726-3 PMC7467845 32878619

[28] Heneghan C (2020) COVID-19: what proportion are asymptomatic? CEBM. https://www.cebm.net/covid-19/covid-19-what-proportion-are-asymptomatic/

[29] World Health Organization (2020) Social stigma threatens COVID-19 response but patients heal faster with everyone's support. World Health Organization Regional Office for Africa. https://www.afro.who.int/news/

[30] Fu L (2020) Clinical characteristics of coronavirus disease 2019 (COVID-19) in China: a systematic review and meta-analysis. Journal of Infection 80, 656–665. 32283155 10.1016/j.jinf.2020.03.041 PMC7151416

[31] Guan W (2020) Clinical characteristics of coronavirus disease 2019 in China. New England Journal of Medicine 382, 1708–1720. 32109013 10.1056/NEJMoa2002032 PMC7092819

[32] Printza A and Constantinidis J (2020) The role of self-reported smell and taste disorders in suspected COVID-19. European Archives of Oto-Rhino-Laryngology 277, 2625–2630. 32447496 10.1007/s00405-020-06069-6 PMC7245504

[33] Chanda-Kapata P (2020) COVID-19 and malaria: a symptom screening challenge for malaria endemic countries. International Journal of Infectious Diseases 94, 151–153. 32344326 10.1016/j.ijid.2020.04.007 PMC7184246

[34] Barranco R and Ventura F (2020) Covid-19 and infection in health-care workers: an emerging problem. The Medico-Legal Journal 88, 65–66. 32441196 10.1177/0025817220923694

